# PP2A Facilitates Porcine Reproductive and Respiratory Syndrome Virus Replication by Deactivating irf3 and Limiting Type I Interferon Production

**DOI:** 10.3390/v11100948

**Published:** 2019-10-15

**Authors:** Jiayu Xu, Lu Zhang, Yunfei Xu, He Zhang, Junxin Gao, Qian Wang, Zhijun Tian, Lv Xuan, Hongyan Chen, Yue Wang

**Affiliations:** 1 State Key Laboratory of Veterinary Biotechnology, Heilongjiang Provincial Key Laboratory of Laboratory Animal and Comparative Medicine, Harbin Veterinary Research Institute, Chinese Academy of Agricultural Sciences, Harbin 150069, China; 2 Department of public health policy, University of California, Irvine, CA 92697, USA

**Keywords:** PRRSV, PP2A, type I interferon, IRF3, antiviral response

## Abstract

Protein phosphatase 2A (PP2A), a major serine/threonine phosphatase in mammalian cells, is known to regulate the kinase-driven intracellular signaling pathways. Emerging evidences have shown that the PP2A phosphatase functions as a bona-fide therapeutic target for anticancer therapy, but it is unclear whether PP2A affects a porcine reproductive and respiratory syndrome virus infection. In the present study, we demonstrated for the first time that inhibition of PP2A activity by either inhibitor or small interfering RNA duplexes in target cells significantly reduced their susceptibility to porcine reproductive and respiratory syndrome virus (PRRSV) infection. Further analysis revealed that inhibition of PP2A function resulted in augmented production of type I interferon (IFN). The mechanism is that inhibition of PP2A activity enhances the levels of phosphorylated interferon regulatory factor 3, which activates the transcription of IFN-stimulated genes. Moreover, inhibition of PP2A activity mainly blocked PRRSV replication in the early stage of viral life cycle, after virus entry but before virus release. Using type I IFN receptor 2 specific siRNA in combination with PP2A inhibitor, we confirmed that the effect of PP2A on viral replication within target cells was an interferon-dependent manner. Taken together, these findings demonstrate that PP2A serves as a negative regulator of host cells antiviral responses and provides a novel therapeutic target for virus infection.

## 1. Introduction

The porcine reproductive and respiratory syndrome virus (PRRSV) is an enveloped, positive-stranded RNA virus, classified in the family Arteriviridae of the order Nidovirales [1,2]. PRRSV is the causative agent of porcine reproductive and respiratory syndrome (PRRS), characterized by respiratory problems in growing pigs and reproductive failure in pregnant sows. There are two major PRRSV groups, based on genetic differences observed in the prototype strains VR2332 (referred to as North American-like strain) and Lelystad (referred to as European-like strain) [3,4]. Although first reported 30 years ago, PRRS remains a severe disease and leads to serious economic losses in the global swine industry [5].

Upon sensing invading viruses, host cells can trigger signaling events that lead to the activation of the innate immune responses. This antiviral response is initiated by the pathogen-associated molecular patterns (PAMPs) through cellular pattern recognition receptors. This induces the production of antiviral molecules such as interferons (IFNs), a broad range of interferon-stimulated genes (ISGs), and inflammatory cytokines [6,7,8]. Interferon regulatory factor 3 (IRF3), a member of the interferon regulatory factors family, plays a central role in type I IFNs induction and antiviral responses [9]. Upon phosphorylation, IRF3 forms a homodimer, followed by translocation to the nucleus where it up-regulates the transcription of type I IFNs [9,10]. The biological activities of type I IFNs are initiated by the binding of IFNα or IFNβ to its cognate receptors on the cell surface [11,12]. The binding of IFNα or IFNβ to its receptors (IFNAR1 and IFNAR2) activates Jak, which phosphorylates the signal transducer and activator of transcription (STAT) proteins, STAT1 and STAT2 [13]. Upon phosphorylation, STAT1 and STAT2 form heterodimers and then recruit IRF9 to form a heterotrimer complex, which translocates to the nucleus and transactivates ISGs to establish an antiviral state [14,15].

It has been reported that target cells recognize the 3′-untranslated region pseudoknot region of the PRRSV genome as PAMPs by RIG-I and TLR3 and induce type I INFs production [16]. To successfully establish productive infection, PRRSV must circumvent the powerful innate immune responses, including those induced by type I IFNs. PRRSV is known to inhibit the synthesis of type I IFNs in infected pigs, and the levels of interferon are not detectable in the lungs of pigs, where PRRSV actively replicates [17,18]. Recent studies have revealed that PRRSV encodes several proteins that act as type I IFNs antagonists to defend against host innate immune responses [18,19,20,21,22,23,24]. For example, PRRSV nsp1α and nsp1β can block the induction of IFNβ at downstream of IRF3 activation [25,26,27,28]. Nsp2, nsp11, and the N protein of PRRSV block type I IFNs production via significantly inhibiting IRF3 phosphorylation and nuclear translocation [29,30]. PRRSV nsp1β and N protein inhibit interferon-activated Jak/STAT signal transduction [31,32]. On the other hand, cellular molecules can also regulate IRF3 activation, such as RACK1 and PP2A, to control the production of type I IFNs [33,34].

PP2A is one of the most ubiquitously expressed and extensively characterized phosphatases in mammalian cells, composed of three different subunits, namely the catalytic subunit, structural scaffold subunit, and regulatory subunit [35]. The phosphatase PP2A is broadly expressed, accounting for a large fraction of phosphatase activity in eukaryotic cells [36]. PP2A activity has broad substrate specificity, thus PP2A plays important roles in modulating cell growth, survival, and apoptosis [37]. Therefore, PP2A has been pointed out to serve as a therapeutic target in various disease models, such as cancer, diabetes, and heart disease [38,39,40]. In spite of the central role of PP2A in many biological processes, how this phosphatase governs PRRSV infection remains unstudied.

In the present study, we investigated the role of PP2A on PRRSV infection. We demonstrate that inhibition of PP2A activity reduces PRRSV replication as a consequence of its phosphatase activity. The interaction between PP2A and IRF3 induced the deactivation of IRF3 and thus blocked type I IFN signaling process. Ablation of PP2A activity using an inhibitor or small interfering RNA (siRNA) stimulated type I IFN signaling pathway and limited PRRSV replication. Further experiments showed that PP2A restricted PRRSV infection at the step of virus replication. Taken together, these data provide the direct evidence that PP2A can dephosphorylate IRF3 and facilitate PRRSV replication.

## 2. Materials and Methods

### 2.1. Cells and Viruses

Primary porcine alveolar macrophages (PAMs) and Marc-145 (a monkey kidney cell line) cells were cultured in Dulbecco’s minimum essential medium (Life Technologies, USA) with 8% heat- inactivated fetal bovine serum (Hyclone, USA), 100 U/mL penicillin, 100 μg/mL streptomycin at 37 °C in a 5% CO_2_ incubator. The PRRSV North American-like strain HuN4 (GenBank accession number EF635006) was grown and titrated in Marc-145 cells as described previously [41] and was stored at −80 °C.

### 2.2. Immunofluorescence Assay 

Immunofluorescence assay (IFA) was performed as described previously with slight changes [42]. Briefly, Marc-145 cells were treated with PP2A inhibitor okadaic acid at indicated concentrations, or transfected with 100 nM control siRNA and PP2Ac specific siRNA duplexes, and followed by infection with PRRSV HuN4 at an multiplicity of infection (MOI) of 0.1 for 24 h. The cells were fixed in 33.3% acetone for 30 min at room temperature. Fixed cells were stained with FITC-conjugated mAb SDOW17 (Rural Technologies, Brookings, USA) to detect PRRSV N protein for 1 h at 37 °C. After three times washing with PBS, the fluorescence was visualized with an Olympus inverted fluorescence microscope equipped with a camera.

### 2.3. Immunoprecipitation and Western Blot 

Samples preparation was performed as previously described with slight modification [43]. In some experiments, cells were lysed with Pierce IP lysis buffer (Thermo Scientific, Rockford, IL, USA). Clarified extracts were first precleared with protein G agarose beads (GenScript, China) and then incubated with protein G agarose beads plus a mAb against PP2Ac (Clone 1D6, Millipore, USA) overnight at 4 °C with gentle rocking. A mouse IgG isotype control plus protein G agarose beads was used as a negative control. The beads were collected by centrifugation, washed with the lysis buffer thrice and resuspended in protein sample buffer. The immunoprecipitated proteins or detergent extracted proteins were separated by SDS-PAGE under reducing conditions and blotted onto polyvinylidene fluoride or polyvinylidene difluoride (PVDF) membranes. The membranes were incubated with the appropriate primary and secondary antibodies, washed, and visualized with an Odyssey instrument (Odyssey infrared imaging system; Li-Cor Biosciences, USA) according to the manufacturer’s instructions. The antibodies against phospho-IRF3 (Ser396) and total IRF3 were obtained from Cell Signaling Technology, USA. Mouse anti-β-actin mAb was purchased from Santa Cruz Biotechnology. Anti-PP2A, C subunit, clone 1D6 is purchased from Millipore. The anti-IFNAR2 was purchased from Sigma. The mouse anti-PRRSV membrane (M) protein mAb was stored in our laboratory.

### 2.4. Quantitative RT-PCR 

Quantitative RT-PCR analyses were carried out as described previously [44]. In brief, total RNA was extracted from cells and subjected to quantitative RT-PCR using gene-specific primers as listed in Table 1. Quantitative reactions were set up in triplicate using SYBR premix Ex Taq (TaKaRa, Japan). Relative quantification was performed by the cycle threshold (ΔΔCT) method [45].

### 2.5. TCID_50_ Assay 

Collected virus samples were clarified by centrifugation at 8000× *g* for 10 min prior to titration. Median tissue culture infectious dose (TCID_50_) assays were performed on Marc-145 cells following the method of Reed and Muench as previously described [46,47]. Briefly, cell monolayers were inoculated with 10-fold serial dilutions of each virus stock and cultured for 6 days prior to observation of the presence of cytopathic effect.

### 2.6. Phosphatase PP2A Inhibition Assay 

To investigate the role of PP2A in PRRSV infection, we used okadaic acid (ApexBio) as a PP2A inhibitor [48]. The cytotoxicity effect of okadaic acid was determined by a cell counting kit-8 (CCK-8) system (Dojindo Laboratory, Kumamoto, Japan) according to the manufacturer’s instructions. In brief, PAMs and Marc-145 cells were cultured in 96-well-plate and incubated with carrier control ethanol or okadaic acid at different concentrations (0, 10, 20, and 40 nM) for 24 h. CCK-8 solution (10 μL per 100 μL of medium in each well) was added, and the plates were then incubated at 37 °C for 3 h. The absorbance of each well was read at 450 nm under a microplate reader. Concentrations of <40 nM were nontoxic (see Results), and cells were therefore incubated with 10 or 20 nM for 1 h before they were inoculated with PRRSV. Unbound virus was removed, and cells were further cultured in the presence of the inhibitor for 24 h to assess the effect of drugs on virus replication.

To confirm PP2A function, PAMs and Marc-145 cells were transfected with 100 nM control siRNA and PP2Ac specific siRNA duplexes (Table 2) using Lipofectamine RNAiMAX reagent (Invitrogen, USA) according to the manufacturer’s instructions. At 24 h post transfection, cells were infected with PRRSV at an MOI of 0.1 or 1.0.

### 2.7. PP2A Phosphatase Activity Assay

Marc-145 cells were treated with various concentrations of PP2A inhibitor okadaic acid or PP2Ac specific siRNA duplexes for 24 h. PP2A phosphatase activity was determined by using ProFluor^®^ Ser/Thr PPase Assay Kit (Promega, USA) as recommended [49]. Briefly, cells were washed with ice-cold 1× DPBS and lysed in the phosphorus-free cell lysis buffer (Beyotime, Nantong, China). PP2Ac subunits were immunoprecipitated from total cell lysates (50 μg) using 3 μg of anti-PP2Ac antibody (Clone 1D6, Millipore) and Protein G agarose beads for 4 h at 4 °C. PP2A catalytic activity was assayed by incubating the immunoprecipitated protein with synthetic phosphorylated bisamide rhodamine 110 peptide substrate at 25 °C for 10 min prior to detection with a Multiscan Spectrum. The PP2A activity data were presented as a percentage of relative PP2A activity compared with control.

### 2.8. Statistical Analysis

All statistical data were expressed as mean ± standard deviation (SD) of three independent experiments and analyzed using Student’s *t*-test. A *p* value of <0.05 was considered statistically significant.

## 3. Results

### 3.1. Pharmacological Inhibition of PP2A Activity Decreases PRRSV Infection

**Figure 1:**
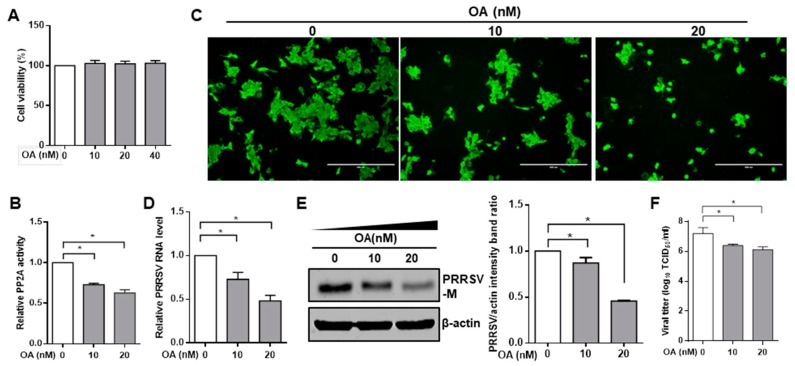
PP2A inhibitor reduces porcine reproductive and respiratory syndrome virus (PRRSV) infection in Marc-145 cells. (**A**) The effect of okadaic acid on Marc-145 cell viability. Marc-145 cells were treated with PP2A inhibitor okadaic acid (OA) at indicated concentrations or the carrier control ethanol for 24 h. Cells were then analyzed with CCK-8 system as described in the Materials and Methods. (**B**) The inhibition effect of okadaic acid on PP2A activity. Marc-145 cells were pre-treated with ethanol or OA (10 nM and 20 nM) for 24 h, PP2A activity was analyzed by Ser/Thr phosphatase kit. (**C**–**F**) Okadaic acid inhibits PRRSV infection and replication in Marc-145 cells. Marc-145 cells were pre-treated with OA or control for 1 h. Cells were then infected with PRRSV at an MOI of 0.1 in the absence or presence of inhibitor. (**C**) At 24 h post infection (hpi), cell monolayers were fixed and examined for virus infection by IFA with an anti-PRRSV N protein mAb. (**D**) Viral RNA levels were determined by quantitative RT-PCR. (**E**) Detergent lysates collected from Marc-145 cells were subjected to immunoblotting with antibodies as indicated, and densitometric data for PRRSV M protein/actin from three independent experiments are expressed as means ± SD. (**F**) Virus titers were detected by TCID_50_ in Marc-145 cells. Results are representative of three independent experiments (means ± SD). *, *p* < 0.05. The *p* value was calculated using Student’s *t*-test.

**Figure 2:**
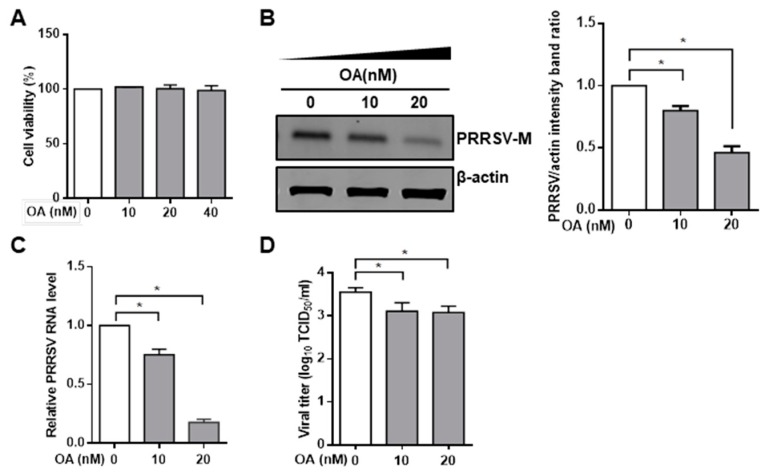
PP2A inhibitor reduces PRRSV infection in PAMs. (**A**) The effect of okadaic acid on PAMs viability. PAMs were treated with ethanol or OA at indicated concentrations for 24 h. Cells were then analyzed with CCK-8 system as described in the Materials and Methods. (**B**–**D**) Okadaic acid inhibits PRRSV infection and replication in PAMs. PAMs were pre-treated with ethanol or OA (10 nM and 20 nM) for 1 h. Cells were then infected with PRRSV at a MOI of 1.0 in the absence or presence of inhibitor. (**B**) At 24 hpi, PRRSV RNA levels were determined by quantitative RT-PCR; (**C**) detergent lysates collected from PAMs were subjected to Western blot with antibodies as indicated, and densitometric data for PRRSV M protein/actin from three independent experiments are expressed as means ± SD; and (**D**) virus samples were collected from cells and measured by TCID_50_. Results are representative of three independent experiments (means ± SD). *, *p* < 0.05. The *p* value was calculated using Student’s *t*-test.

To investigate the effect of PP2A on PRRSV infection, we exposed cells to pharmacological drug okadaic acid, which is known to be an inhibitor of protein phosphatase PP2A [50,51]. The cytotoxic effect of okadaic acid was examined by CCK8 in Marc-145 cells and PAMs. No significant toxicity of okadaic acid was evident for the two types of cells at concentrations of <40 nM (Figure 1A and Figure 2A). The inhibitory effect of okadaic acid on PP2A activity was also confirmed by a PP2A phosphatase assay kit in Marc-145 cells (Figure 1B). Thus, Marc-145 cells were treated with PP2A inhibitor okadaic acid for 1 h, and cells were then inoculated with PRRSV. The IFA showed that the number of PRRSV-positive cells was dramatically lower in okadaic acid-treated cells than that in mock control (Figure 1C). Marc-145 cells treated with okadaic acid reduced viral RNA levels (Figure 1D) and viral protein synthesis (Figure 1E) in a concentration-dependent manner. We also used the TCID_50_ assay to evaluate the effect of the inhibitor on PRRSV yield, and the results again showed that that okadaic acid treatment decreased the levels of virus titers (Figure 1F). Consistent with these findings obtained with Marc-145 cells, PP2A inhibitor okadaic acid had similar inhibitory effect on PRRSV infection in PAMs (Figure 2B–D). These data suggest that inhibition of phosphatase PP2A activity results in the decrease of PRRSV infection.

### 3.2. Knockdown of Endogenous PP2Ac Expression Reduces PRRSV Infection 

**Figure 3:**
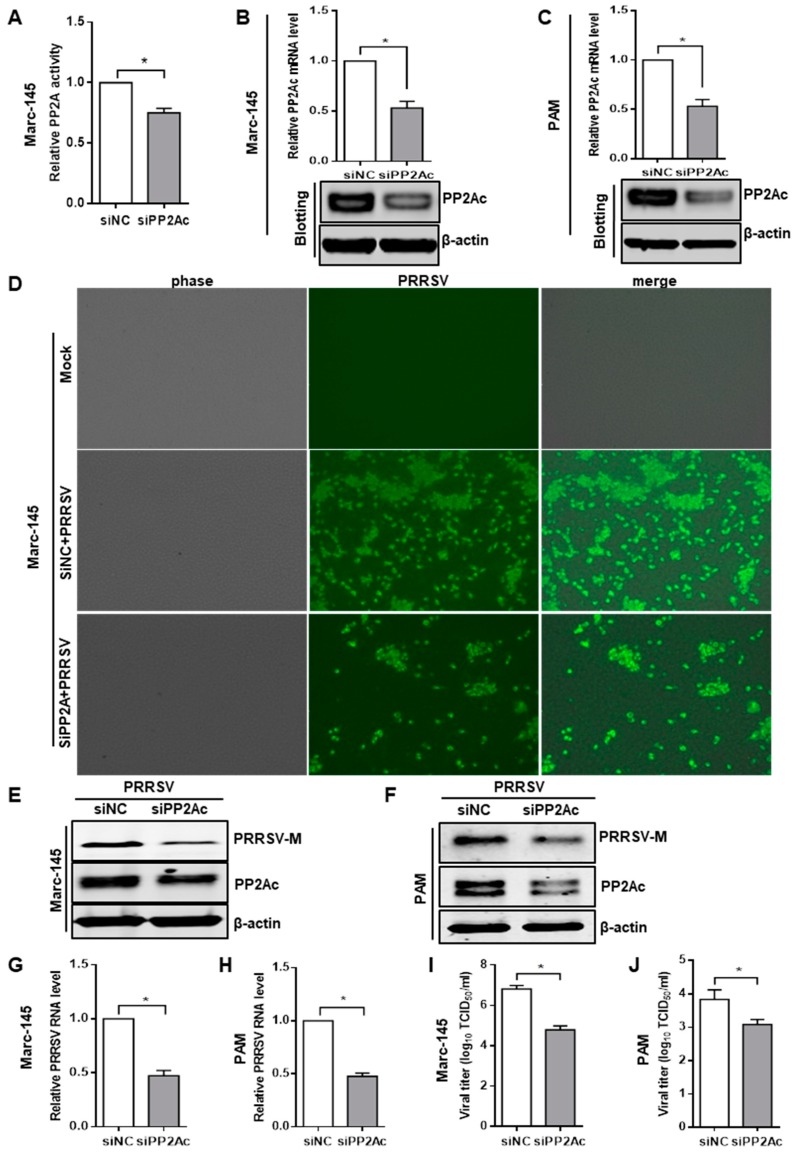
Knockdown of the endogenous PP2Ac expression decreases PRRSV infection. (**A**) Verification of PP2Ac specific siRNA on PP2A activity. Marc-145 cells were transfected with PP2Ac specific siRNA (siPP2Ac) or negative control siRNA (siNC) for 24 h, relative PP2A activity was analyzed. (**B**,**C**) PP2Ac siRNA reduced endogenous PP2Ac expression. Marc-145 cells (B) and PAMs (C) were transfected with siPP2Ac or siNC for 24 h, and the knockdown efficiency of PP2Ac was determined by quantitative RT-PCR and western blot. (**D**–**J**) Knockdown endogenous PP2Ac expression inhibits PRRSV infection. Cells were transfected with siPP2Ac or siNC for 24 h, and cells were then exposed to PRRSV for 24 h. Cell monolayers were fixed and examined for virus infection by IFA (200×) (**D**); viral M protein levels in Marc-145 cells (**E**) and PAMs (**F**) treated with siRNA were analyzed by Western blot; the levels of viral RNA in Marc-145 cells (**G**) and PAMs (**H**) were determined by quantitative RT-PCR; and virus titers in Marc-145 cells (**I**) and PAMs (**J**) were evaluated with TCID_50_. Results are representative of three independent experiments (means ± SD). *, *p* < 0.05. The *p* value was calculated using Student’s *t*-test.

Having found that PRRSV infection is inhibited by phosphatase PP2A inhibitor okadaic acid, we attempted to genetically modify PP2A expression to confirm its effect. The PP2A core enzyme is composed of a scaffold subunit A, a regulatory subunit B, and a 36 kDa catalytic subunit C (PP2Ac) [52], and evidences demonstrated that modifying PP2Ac alone could adjust PP2A function thus to alter its substrates activation [53,54,55]. Therefore, siRNA duplexes were used to knockdown the endogenous expression of PP2Ac. When Marc-145 cells were transfected with PP2Ac siRNA, the PP2A activity was significantly decreased relative to that in cells transfected with control siRNA (Figure 3A). Western blot and quantitative RT-PCR analysis of cell lysates collected from Marc-145 cells transfected with PP2Ac siRNA revealed a clear reduction in the levels of PP2Ac RNA and protein (Figure 3B). At 24 h post siRNA transfection, Marc-145 cells were inoculated with PRRSV for an additional 24 h. We observed that PRRSV-positive cells were considerably lower in PP2Ac siRNA-treated cells than that in control siRNA (Figure 3D). The levels of virus protein were greatly decreased in PP2Ac siRNA transfection group compared with the control siRNA group (Figure 3E). Knockdown of endogenous PP2Ac with siRNA also consequently reduced virus loads as measured by quantitative RT-PCR and TCID_50_ (Figure 3G,I). Consistent with the results in Marc-145 cells, we found that PP2Ac specific siRNA significantly decreased the levels of virus protein, viral RNA and virus titers in PAMs as well (Figure 3C,F,H,J). Taken together, these data demonstrate that PP2A activity positively regulated PRRSV infection.

### 3.3. Ablation of Endogenous PP2A Activity Enhances Antiviral Responses in Target Cells 

**Figure 4:**
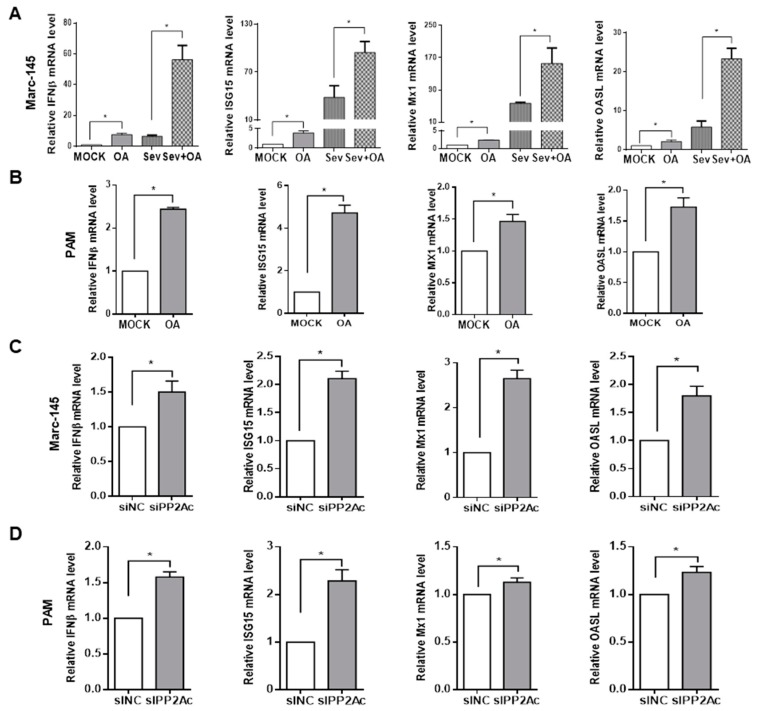
Reduction of PP2A activity enhances the mRNA levels of interferon β (IFNβ) and some interferon-stimulated genes (ISGs). (**A**) Marc-145 cells were treated with PP2A inhibitor OA at 15 nM for 24 h and followed by the infection with SeV for 12 h. (**B**) PAMs were treated with PP2A inhibitor OA at 15 nM for 24 h. Total RNA was extracted, and the RNA levels of IFNβ, ISG15, Mx1, and OASL were determined by quantitative RT-PCR. Marc-145 cells (**C**) and PAMs (**D**) were transfected with control siRNA or PP2Ac siRNA for 24 h, respectively. Total RNA was extracted, and the RNA levels of IFNβ, ISG15, Mx1, and OASL were determined by quantitative RT-PCR. Results are representative of three independent experiments (means ± SD). *, *p* < 0.05. The *p* value was calculated using Student’s t-test.

Given the fact that inhibition of PP2A activity can decrease PRRSV infection, it is worthwhile to further investigate the molecular mechanisms. Type I IFNs, or IFNα and IFNβ are the critical innate immune cytokines in controlling antiviral function [56], thus we next investigated whether destruction of PP2A function promotes type I IFNs antiviral activity. Marc-145 cells and PAMs were treated with okadaic acid for 24 h, and some Marc-145 cells were further infected with Sendai virus (Sev) for another 12 h, the mRNA levels of IFNβ and several ISGs including ISG15, Mx1, and OASL were then analyzed [57]. It was shown that the mRNA levels of IFNβ, ISG15, Mx1, and OASL were significantly increased in okadaic acid treated Marc-145 cells (Figure 4A) and PAMs (Figure 4B) in contrast to the control treatments in the absence or the presence of SeV, suggesting that inhibition of PP2A activity enhances type I IFNs signaling pathway in the resting cells. Similar outcomes were also observed in PP2Ac specific siRNA treated Marc-145 cells (Figure 4C) and PAMs (Figure 4D). These results illustrate that suppression of PP2A function up-regulates type I IFNs signaling pathway in target cells.

### 3.4. PP2A Dephosphorylation Activity Deactivates IRF3 and Limits Type I IFN Signaling

**Figure 5:**
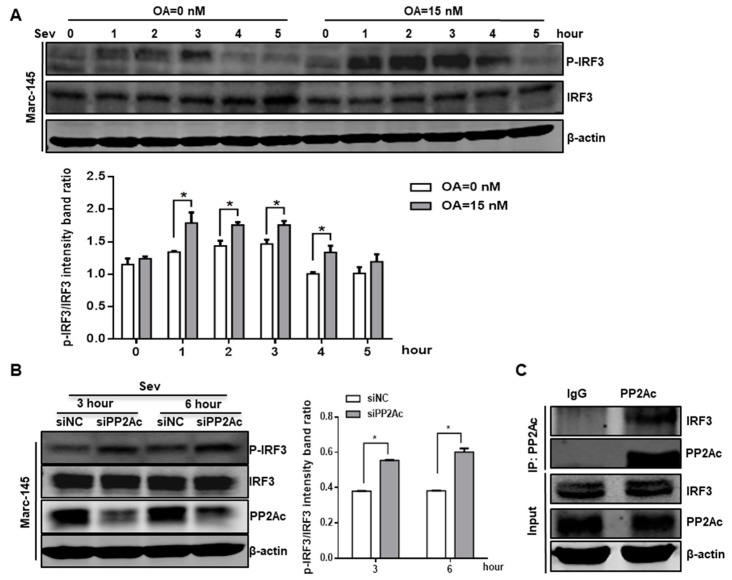
Interaction between PP2Ac and IRF3 decreases the phosphorylation levels of IRF3. (**A**) Marc-145 cells were treated with either carrier control ethanol or PP2A inhibitor OA at a concentration of 15 nM for 24 h. Cells were infected with SeV for indicated times, and were then directly subjected to Western blot with antibodies against phosphorylated IRF3 (P-IRF3), total IRF3, and β-actin. (**B**) Marc-145 cells were transfected with siPP2Ac or siNC for 24 h, and followed by SeV infection. Detergent lysates collected from SeV-stimulated cells were blotted with antibodies as indicated. Densitometric data for P-IRF3/ IRF3 from three independent experiments are expressed as means ±SD. (**C**) Marc-145 cells were harvested, and immunoprecipitation and Western blot were performed as described in the Materials and Methods to examine interactions between PP2Ac and IRF3. *, *p* < 0.05. The *p* value was calculated using Student’s *t*-test.

The interferon regulatory factor family, like IRF3 protein, is a group of transcription factors that play pivotal roles in many aspects of the immune response [57]. Previous studies have shown that IRF3 plays a central role in type I IFNs induction and antiviral responses [58,59,60,61]. Transcriptional activity of IRF3 is controlled by virus-induced C-terminal phosphorylation events on serines 385, 386, and 396, as well as the threonine 405 [9,62]. Here, we evaluated whether the role of PP2A in type I IFNs induction attributes to IRF3 activation by using a polyclonal antibody against phospho-IRF3 (Ser396). Marc-145 cells were pretreated with okadaic acid, and cells were then inoculated with SeV, which can activate IRF3 during the course of infection [63]. As shown in Figure 5A, okadaic acid treatment potentiated phosphorylation of IRF3 at Ser396 in response to SeV infection, thereby supporting an inhibition function of PP2A in virus-induced IRF3 regulation. Consistently, we observed a higher level of phosphorylated IRF3 in response to combined treatment of SeV infection and PP2Ac siRNA (Figure 5B). These data suggest that PP2A functions as a negative regulator of type I IFN signaling by deactivating IRF3.

Next, we investigated the possibility that PP2A interacts with IRF3 and deactivates IRF3 in Marc-145 cells. An endogenous co-immunoprecipitation assay was performed to explore the interaction between PP2A and IRF3. Endogenous IRF3 was efficiently co-immunoprecipitated by anti-PP2Ac antibody but not by the isotype control IgG in Marc-145 cells (Figure 5C), suggesting that PP2A maintained low IRF3 phosphorylation in the resting Marc-145 cells. Collectively, these results indicate that the PP2A-IRF3 interaction deactivated IRF3 and led to the decline of type I IFN signaling.

### 3.5. PP2A Plays a Critical Role in the Control of PRRSV Replication

**Figure 6:**
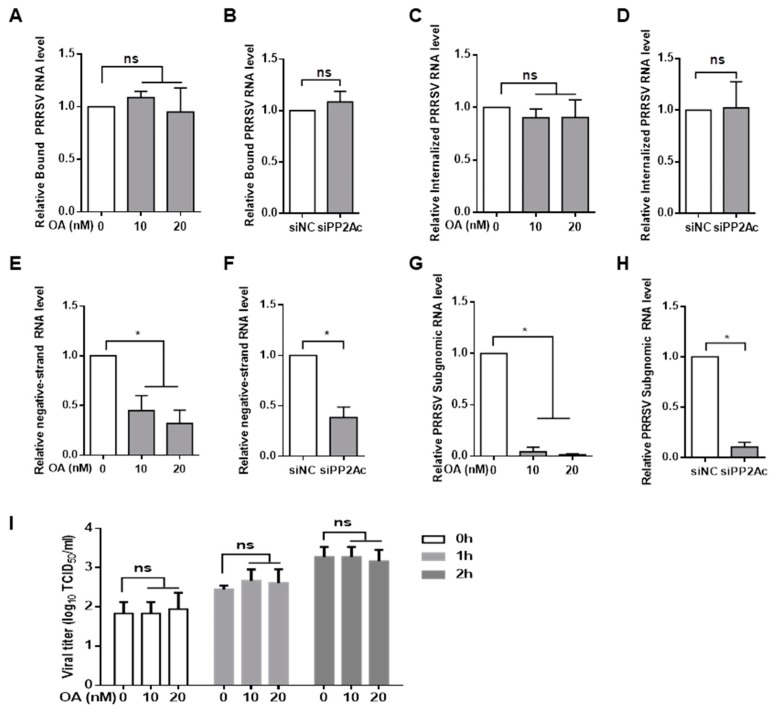
Ablation of PP2A activity restricts PRRSV replication. (**A**,**B**) Inhibition of PP2A activity does not affect PRRSV attachment. (**A**) Marc-145 cells were pre-treated with OA for 1 h; (**B**) Marc-145 cells were transfected with siNC or siPP2Ac for 24 h. Cells were then incubated with PRRSV at an MOI of 5.0 for 2 h at 4 °C. Unbound viral particles were removed by washing thoroughly with ice-cold PBS five times. Total RNA was extracted, and PRRSV RNA levels were analyzed by quantitative RT-PCR. (**C**,**D**) Inhibition of PP2A activity does not affect PRRSV internalization. After washing with ice-cold PBS as described above, the cell monolayers were further cultured for 2 h in the presence of OA at 37 °C (C) or cells were then cultured in fresh media at 37 °C for 2 h (D). Internalized viral RNA levels were determined by quantitative RT-PCR. (**E**–**H**) Inhibition of PP2A activity affects PRRSV replication within the cells. After treatment with OA for 1 h, Marc-145 cells were infected with PRRSV at an MOI of 1.0 for 1 h and were then cultured for an additional 8 h in the presence of OA. Total RNA was extracted, and the relative levels of PRRSV negative-strand RNA (**E**) and subgenomic RNA (**G**) were determined by quantitative RT-PCR. Marc-145 cells were transfected with siNC or siPP2Ac for 24 h, and cells were then infected with PRRSV at an MOI of 1.0 for 8 h. The relative levels of PRRSV negative-strand RNA (**F**) and subgenomic RNA (**H**) were determined. (**I**) PP2A does not affect PRRSV release. Marc-145 cells were infected with PRRSV at an MOI of 1.0. At 8 hpi, the media containing virus particles were removed and replaced with fresh medium in the presence of OA as indicated. Culture media were harvested at 0, 1, and 2 h, and titrated by TCID_50_. Results are representative of three independent experiments (means ± SD). *, *p* < 0.05. The *p* value was calculated using Student’s *t*-test.

Since inhibition of PP2A activity significantly blocked PRRSV infection by triggering IRF3- mediated type I IFNs induction, experiments were designed to identify the step of the virus life cycle that was associated with the PP2A-mediated signaling pathway. To assess whether inhibition of PP2A activity affected PRRSV attachment to cells, Marc-145 cells were pretreated with okadaic acid and exposed to PRRSV at 4 °C to allow binding but prevent internalization. After extensive washing, bound virus was detected by quantitative RT-PCR assay, and we observed that pretreatment with inhibitor had no effect on virus binding (Figure 6A). Similarly, PP2Ac knockdown did not affect virus attachment to Marc-145 cells as measured by quantitative RT-PCR (Figure 6B), indicating that inhibition of PP2A activity had no effect on PRRSV attachment to target cells.

We next determined whether PP2A affected virus internalization. Marc-145 cells were pretreated with okadaic acid or the carrier control and exposed to PRRSV at 37 °C. After two hours, the unbound virus was removed by extensive washing, and cellular total RNA was extracted for virus RNA quantification. Treatment with okadaic acid did not alter the amount of viral RNA in contrast to control treatment (Figure 6C). There was no difference in the amount of viral RNA that was associated with PP2Ac siRNA-treated cells compared with the control siRNA group (Figure 6D), suggesting that inhibition of PP2A activity had no effect on virus penetration.

Since it appeared that inhibition of PP2A activity did not block virus binding and entry, we questioned whether inhibition of PP2A restricted virus biosynthesis. It was shown that pretreatment of Marc-145 cells with okadaic acid significantly inhibited the synthesis of negative-strand viral RNA in contrast to the carrier control (Figure 6E). Furthermore, the presence of okadaic acid obliterated the accumulation of viral subgenomic RNA within the cells (Figure 6G). Consistent with the results obtained with okadaic acid, PP2A siRNA significantly decreased the levels of viral negative-strand RNA and subgenomic RNA as well (Figure 6F,H). These data indicated that PRRSV was not able to replicate efficiently within the host cell when PP2A activity was inhibited.

Furthermore, we evaluated whether inhibition of PP2A activity elicited virion release. Marc-145 cells were infected with PRRSV for 8 h, and the inoculum was then replaced by fresh medium containing okadaic acid. Supernatants were collected at indicated times and titrated by TCID_50_ assay. There was no significant difference in virion release between cells treated with okadaic acid and cells treated with control (Figure 6I). Taken together, these data indicated that inhibition of PP2A activity reduced PRRSV infection at the viral replication step but not at the binding, penetration, and releasing steps of viral life cycle.

### 3.6. Inhibition of PP2A Activity Suppresses PRRSV Replication via Type I IFN Signaling

**Figure 7:**
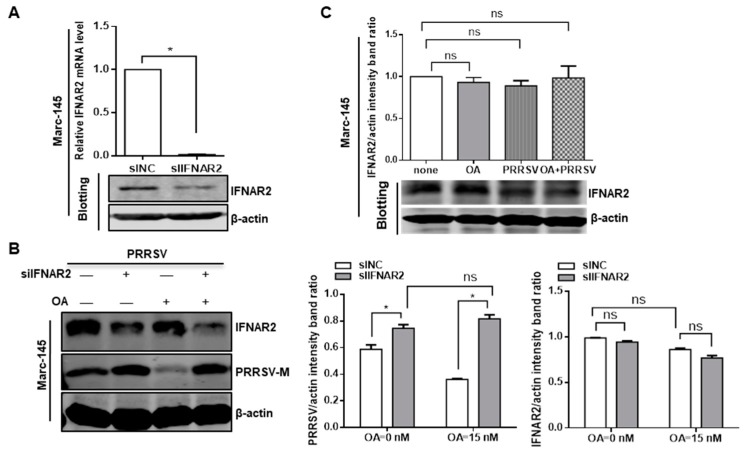
Inhibition of PP2A activity suppresses PRRSV replication via type I IFN pathway. (**A**) Marc-145 cells were transfected with IFNAR2 specific siRNA or siNC for 24 h, and the knockdown efficiency of IFNAR2 was determined by quantitative RT-PCR and western blot. (**B**) Marc-145 cells were transfected with siNC or IFNAR2 siRNA for 24 h and then were treated with ethanol or OA (15 nM) for 1 h. Cells were then infected with PRRSV in the presence of OA. At 24 hpi, detergent lysates collected from cells were subjected to Western blot with antibodies as indicated. Densitometric data for PRRSV M protein/actin and IFNAR2 protein/actin from three independent experiments are expressed as means ± SD. (**C**) Marc-145 cells were treated with OA at 15 nM or ethanol for 24 h. Some Marc-145 cells were pre-treated with OA or control for 1 h, and cells were then infected with PRRSV at an MOI of 1.0 in the presence of inhibitor. At 24 hpi, detergent lysates collected from Marc-145 cells were subjected to immunoblotting. Densitometric data for IFNAR2 protein/actin from three independent experiments are expressed as means ± SD. Results are representative of three independent experiments (means ± SD). *, *p* < 0.05. The *p* value was calculated using Student’s *t*-test.

To confirm the effect of PP2A on PRRSV replication is through type I IFNs but not through the virus itself, the relationship between PP2A and virus was determined. Type I IFNs engage their cognate receptors IFNAR1 and IFNAR2, to activate their downstream signaling pathway and exert antiviral responses [64,65]. Therefore, we used an IFNAR2 specific siRNA to knockdown the endogenous expression of IFNAR2. A clear reduction of IFNAR2 mRNA and protein levels was observed (Figure 7A), indicating that IFNAR2 siRNA had functioned properly. Under these conditions, we assessed the effect of the interaction between PP2A and type I IFN signaling on PRRSV replication. Marc-145 cells were first transfected with control siRNA or IFNAR2 specific siRNA for 24 h, and the cells were then treated with okadaic acid for 1 h. The cells were followed by incubation with PRRSV for an additional 24 h in the presence of okadaic acid, and cell lysates were collected for Western blot analysis. The results showed the okadaic acid treatment blocked viral protein synthesis and IFNAR2 siRNA facilitated the levels of viral protein, whereas the decrease of viral protein synthesis by okadaic acid was blocked by depletion of IFNAR2 expression (Figure 7B). In the control treatments, the relative IFNAR2 levels with mock and PRRSV infection did not significantly impact IFNAR2 expression in the presence or absence of okadaic acid (Figure 7C). Collectively, the mechanism that PRRSV replication was blocked by the inhibition of PP2A function was due to type I IFNs-mediated antiviral signaling pathway but not through impairing virus itself.

## 4. Discussion

Emerging evidences indicate that PP2A phosphatase activity plays a critical role in cellular functions [33,35,66,67]. However, detailed mechanisms of this phosphatase involved in virus infection have not been carefully studied. In the present study, we reported for the first time that cellular phosphatase PP2A positively regulated PRRSV infection. The molecular mechanism was that PP2A inhibits type I IFN signaling pathway through dephosphorylating IRF3, thereby facilitating viral replication.

Phosphorylation and its counterpart dephosphorylation are critical for protein functions by detaching or attaching phosphoric esters and anhydrides. Many enzymes, receptors, and signaling cascades are switched “on” or “off” by phosphorylation and dephosphorylation [68]. PP2A is one of the most abundant protein phosphatases used in dephosphorylation in eukaryotes [69,70]. Down- regulation of PP2A activity is reported in some diseases such as cancer and neurodegenerative disorders, which indicates the involvement of PP2A in these disorders [71,72,73]. Some researchers have found that virus infection can also affect PP2A activity to result in severe disease development such as HBV and HCV [74,75,76,77]. Therefore, PP2A has been recognized as a therapeutic target in various disease models [40]. Here, PRRSV infection does not significantly affect PP2A activity and PP2A expression levels (data not shown), indicating that the virus does not result in the loss of PP2A function. However, when using PP2A inhibitor okadaic acid, we observed that the PRRSV infection was blocked. Although okadaic acid has been proved to inhibit PP2A activity among a wide range of cells [78,79], siRNA was also used to specifically knockdown the expression of PP2Ac to confirm the impact of PP2A on PRRSV infection. To further confirm the involvement of PP2A in a virus infection, we tried to overexpress the fully active PP2Ac to increase PP2A activation, but there was no significant effect (data not shown). Heretofore attempts to overexpress PP2Ac also failed [70,80]. The reasonable interpretation is that PP2A is one of the most abundant enzymes, accounting for up to 1% of total cellular protein in some tissues, so its expression and activity is tightly regulated. Our data obtained from both inhibitor and genetic modifications altogether suggest that PP2A phosphatase was involved in PRRSV infection.

During virus infection, the innate immune response is often activated, leading to the induction of type I IFNs, which is pivotal for the cellular antiviral responses [68,81,82]. Here, we observed that inhibition of PP2A can initiate type I IFN signaling cascades. After viral infection, pattern recognition receptors detect viral molecular features and activate the essential transcriptional regulator IRF3 via phosphorylation of its C-terminal trans-activation domain by TBK1/IKKε, and trigger the production of type I IFNs [83,84]. However, excessive IRF3 activation and type I IFNs production could cause autoimmune disease such as STING-associated vasculopathy of infancy [85,86]. In this study, we observed that type I IFNs signaling pathway regulated by phosphatase PP2A is IRF3-dependent, suggesting that the PP2A dephosphorylates active IRF3, thus turning off type I IFN signaling. Co-immunoprecipitation assay revealed that in resting cells PP2A interacts with IRF3 and controls the phosphorylation level of IRF3 as thus it can limit excessive innate immune response and balance cellular function. Similarly, some researchers report that PP2A can recognize and dephosphorylate the active IRF3, which thus shutting off the transcriptional signal and interferon synthesis [33,34]. Recently, Peng et al. found that the phosphatase PP2A is recruited by the adaptor FBXO17 to dephosphorylate IRF3 and negatively regulates the type I IFN signaling pathway [87]. Here, we demonstrated that PP2A played a vital role in controlling IFNs signaling by regulating the IRF3 phosphorylation level during PRRSV infection.

It is clear that type I IFNs can induce transcription of a significant number of genes that mediate the antiviral state in target cells [76]. The dissemination of virus infection throughout a host is the consequence of multiple processes that include replication of viral nucleic acids, protein synthesis, and cell–cell spread of infectious entities [88]. Here, we examined how the phosphatase PP2A affects virus infection by tracking down the four steps of viral replication cycle: attachment, penetration, replication, and release. Our results demonstrated that inhibition of PP2A reduced PRRSV infection at the step of viral replication rather than viral attachment, penetration, or release. Emerging shreds of evidence have shown that PRRSV has evolved different strategies to subvert type I IFN signaling pathway, see reviews [23,89]. However, the antiviral activity of type I IFNs against PRRSV infection remains unclear, here we demonstrated that the deactivated IRF3 by PP2A inhibited the type I IFN signaling pathway and facilitated the viral replication step during virus infection.

PP2A is a multimeric holoenzyme composed by a scaffold A, a regulator B, and a catalytic C subunit, and its substrate specificity is determined by the regulatory B subunits [69,70,72,90,91]. PP2A has been reported to interact with more than 50 proteins, thus regulating many cell functions including other transcription factors, such as NF-κB and p53 [92,93]. A recent study reports that Ebola virus transcription factor VP30 is dephosphorylated by the phosphatase PP2A, which suppresses virus transcriptions and infection [94]. Hence, we examined whether PP2A can directly affect the function of viral proteins during PRRSV infection. We observed that the inhibition effect of okadaic acid on viral replication was not recovered by IFNAR2 knockdown treatment. These data suggest that the regulatory role of PP2A in PRRSV replication was through the IRF3-mediated type I IFN signaling pathway but not through direct dephosphorylation of viral proteins.

In summary, here we have examined the interaction between phosphatase PP2A and type I IFN signaling pathways in the regulation of PRRSV infection. Our findings have shown that PP2A positively regulated PRRSV replication through dephosphorylating active IRF3 via direct interaction, which suppressed the antiviral activity of type I interferon. Although the direct interaction between PP2A and PRRSV proteins remained to be elucidated, we provided indirect evidence that the regulatory role of PP2A on virus replication was type I IFNs dependent. Together, we uncovered a novel mechanism that PP2A served as a negative regulator of host cell antiviral responses, which might be valuable for understanding PRRSV-related pathogenesis.

## Figures and Tables

**Table 1:** Primers used for relative quantitative RT-PCR.

Primer Name	Sequence (5′-3′)
PRRSV-F	AGATCATCGCCCAACAAAAC
PRRSV-R	GACACAATTGCCGCTCACTA
PRRSV-subgenomic-F	GCCCAAAACTTGCTGCACG
PRRSV-subgenomic-R	GACACAATTGCCGCTCACTA
Porcine –β-actin-F	CTTCCTGGGCATGGAGTCC
Porcine –β-actin-R	GGCGCGATGATCTTGATCTTC
Porcine-IFNβ-F	GCTAACAAGTGCATCCTCCAAA
Porcine-IFNβ-R	CCAGGAGCTTCTGACATGCCA
Porcine-ISG15-F	GATGCTGGGAGGCAAGGA
Porcine-ISG15-R	CAGGATGCTCAGTGGGTCTCT
Porcine-Mx1-F	GAACGAAGAAGACGAATGGAAGG
Porcine-Mx1-R	GATGCCAGGAAGGTCTATGAGG
Porcine-OASL-F	GAAGAATGTGCAGGTGCTAGAGG
Porcine-OASL-R	GATGCCAGGAAGGTCTATGAGG
Marc145-Mx1-F	CATCACTGCTCTCATACAAGGGG
Marc145-Mx1-R	CCATTTGTGGAACTCACGTCG
Marc145-OASL-F	ACGAATAGTGAGTGTGAGGGC
Marc145-OASL-R	ATGGAAGGGACTCACTCACG
Marc145-ISG15-F	CACCGTGTTCATGAATCTGC
Marc145-ISG15-R	CTTTATTTCCGGCCCTTGAT
Marc145-actin-F	ATCGTGCGTGACATTAAG
Marc145-actin-R	ATTGCCAATGGTGATGAC
Marc145-IFNβ-F	TGCTCTCCTGTTGTGCTTCTC
Marc145-IFNβ-R	CTGCGGCTGCTTAATTTCCTC
Marc145-IFNAR2-F	TGAACCGCCAGAGTTTGAGA
Marc145-IFNAR2-R	ACACAGTAGTTTGCATTTGGAAT

**Table 2:** Sequences of sense strand of siRNA against target gene in porcine alveolar macrophage (PAM) or Marc-145 cells.

Target	Sense Strand Sequence (5′–3′)
PP2Ac siRNA	GCUCGUGAUGGAGGGAUAUTT
IFNAR2 siRNA	GCCAGAGUUUGAGAUUGUUTT
Control siRNA	UUCUCCGAACGUGUCACGUTT
